# Veterinary Preparatory Education

**Published:** 1898-08

**Authors:** Robert Robb

**Affiliations:** Littleton, Iowa


### VETERINARY PREPARATORY EDUCATION.

By Robert Robb, V.S., M.D.,
LITTLETON, IOWA.

Much has been said of veterinary education in the various journals, but somehow the preparatory condition of the student has been omitted, so it is with pleasure that I relate some of the conditions necessary for the veterinary student before he attempts to enter college. It is not my intention to go into the details of the curriculum of the various colleges all over the land, but to point out some of the important factors that are so essential to students in after-life that go to make up a practitioner.

At the present day I may say there are so many practitioners who are so handicapped in their profession that it is embarrassing to witness them in their routine work. In the first place, students generally enter college with very little knowledge of their profession; they go unprepared, so to speak, thinking that by accomplishing anatomy, physiology, pathology, materia medica, practice, and bacteriology they have studied the veterinary art to perfection. I may say, without any contradiction, that the most important place for study is in the stable. Few have the early training as to the management of stock, including breeding, feeding, grooming, shoeing, and the general care of veterinary patients. A few go from the farm and other establishments prepared with the above knowlege, while others who have been unfortunate to such surroundings may in time attain a fair knowledge of the rudiments, while a very large majority leave the bank, store, butchershop, unable to harness or hook up a horse, or even to know when one is properly hooked, not alone to speak of other things purporting to the general management of stock. Such men do not know the habits of healthy animals, studying only the habits of sick animals.

I must admit, with a few of my brethren, that it is hard for any practitioner to diagnose between health and disease if he is not accustomed to the habits and different traits that animals are prone to both in health and in sickness. This drawback among veterinarians in America has been one of the greatest curses in the country to damage the veterinary profession—men with diplomas, with fair education, while others well-learned have gone out as honest, conscientious practitioners only to stumble up against little things that they thought unnecessary for them to know or learn, having never been taught that the study and science of little things was the study of big things and the key to advancement. I am convinced that it is impossible for any man to be a successful practitioner unless he has been raised up to the profession or born in the stable, and, as age advances, having the necessary surroundings that will lead him to witness the different cases presented for examination both in health and in disease. By so doing he learns to diagnose lameness, habits, conformation, and disease. The ability to diagnose lameness in all its phases is only acquired by long experience and observation. This branch is one of the greatest obstacles that 80 per cent. fail in during their routine practice.

There is no question that some men are gifted with this talent; in fact, it comes to them as natural as if going to their meals, while others could not tell whether it was in front or behind unless the animal was dead lame, and then they might fear it was in the cerebrum. If students that, by natural instinct, are adapted to the profession could begin early and start at the bottom—first in the stable, then the shoeing-shop, then the college—and accomplish the end they have in view, what a blessing it would be to the profession and dumb brutes. They could diagnose their cases accurately, give their prognosis without a doubt, and give better satisfaction to all concerned, and by so doing they would be able to advance veterinary science so that it would have a better foothold both in America and foreign countries.

At the present day students desire to become doctors in two or three years, experts in judging soundness, conformation, and lameness. I wonder where they expect to be taught all this. It is very hard to make a veterinarian out of a man unless he has been born one, either in the stall or manger; "either place will do." This old adage proves itself every day in the eyes of those whose observations are the keenest.

In all the cities how many veterinarians can remove a shoe, dress a foot, and drive a shoe properly? Are there 5 per cent., or may I say 2 per cent.? Every practitioner should be able to do this independently of any person. By so doing he could be able to cope with the few who exist. One must feel like taking a back seat every time he has to send his patient to a shoeing-shop, not speaking of the inconvenience, to have a shoe removed or foot examined, or a shoe put on. How professional it would look, and at the same time advance the profession, if all the veterinarians could remove, dress, and clinch.

To recapitulate the course for veterinary students before entering college, I would say acquire the knowledge of diagnosing disease and lameness to a certain extent, spend two years in a thorough shoeing-establishment; then you will feel as if you are somebody, fit for your professional career, should you be able to pass your final examination.

